# Integrated analysis of pivotal biomarker of LSM1, immune cell infiltration and therapeutic drugs in breast cancer

**DOI:** 10.1111/jcmm.17436

**Published:** 2022-06-12

**Authors:** Yen‐Dun Tony Tzeng, Kuan‐Hao Tsui, Ling‐Ming Tseng, Ming‐Feng Hou, Pei‐Yi Chu, Jim Jinn‐Chyuan Sheu, Chia‐Jung Li

**Affiliations:** ^1^ Department of Surgery Kaohsiung Veterans General Hospital Kaohsiung Taiwan; ^2^ Institute of Biomedical Sciences National Sun Yat‐sen University Kaohsiung Taiwan; ^3^ Department of Obstetrics and Gynecology Kaohsiung Veterans General Hospital Kaohsiung Taiwan; ^4^ Institute of BioPharmaceutical Sciences National Sun Yat‐sen University Kaohsiung Taiwan; ^5^ School of Medicine National Yang‐Ming University Taipei Taiwan; ^6^ Comprehensive Breast Health Center Taipei Veterans General Hospital Taipei Taiwan; ^7^ Division of Breast Surgery, Department of Surgery, Center for Cancer Research Kaohsiung Medical University Chung‐Ho Memorial Hospital Kaohsiung Taiwan; ^8^ Department of Pathology Show Chwan Memorial Hospital Changhua Taiwan

**Keywords:** breast cancer, diagnosis, immune infiltration, LSM1, multi‐omics

## Abstract

The discovery of early diagnosis and prognostic markers for breast cancer can significantly improve survival and reduce mortality. LSM1 is known to be involved in the general process of mRNA degradation in complexes containing LSm subunits, but the molecular and biological functions in breast cancer remain unclear. Here, the expression of LSM1 mRNA in breast cancer was estimated using The Cancer Genome Atlas (TCGA), Oncomine, TIMER and bc‐GenExMiner databases. We found that functional LSM1 inactivation caused by mutations and profound deletions predicted poor prognosis in breast cancer (BRCA) patients. LSM1 was highly expressed in both BRCA tissues and cells compared to normal breast tissues/cells. High LSM1 expression is associated with poorer overall survival and disease‐free survival. The association between LSM1 and immune infiltration of breast cancer was assessed by TIMER and CIBERSORT algorithms. LSM1 showed a strong correlation with various immune marker sets. Most importantly, pharmacogenetic analysis of BRCA cell lines revealed that LSM1 inactivation was associated with increased sensitivity to refametinib and trametinib. However, both drugs could mimic the effects of LSM1 inhibition and their drug sensitivity was associated with MEK molecules. Therefore, we investigated the clinical application of LSM1 to provide a basis for sensitive diagnosis, prognosis and targeted treatment of breast cancer.

## 1 INTRODUCTION

In the past few years, breast cancer research has become a rapidly developing field worldwide. Carcinogenic effects are multifactorial involving multiple factors such as genetics, environment or ageing. Recent research to elucidate the biological and molecular pathways of tumours that mediate cancer progression and drug resistance has led to the development of various molecularly targeted therapies, including monoclonal antibodies, small molecule receptor tyrosine kinase inhibitors and drugs that block downstream signalling pathways in breast cancer.^1^ Breast cancer has become a prominent example of the success of precision medicine in the treatment of solid tumour malignancies.^2^ The first step in this process involves new blood‐based diagnostics that can now provide clinically useful information in a non‐invasive manner. However, there is an urgent need to identify novel biomarkers that can be used for early diagnosis, particularly to guide initial therapy and to predict relapse or resistance after novel targeted therapies.^3^

LSM1, also known as CaSm (cancer‐associated Sm‐like), is a family of Sm proteins that were first discovered during the study of human precursor RNA processing, and is a family of highly conserved homologous proteins containing Sm motifs, so‐called Sm‐like (LSm) proteins.^4^ LSM1 was originally identified by its elevated expression in pancreatic cancer‐derived cell lines. LSM1 expression leads to increased growth, decreased chemosensitivity and enhanced migration/invasion of pancreatic cancer cells.^4^, ^5^, ^6^ The upregulation of LSM1 alters the expression of genes critical mediators of apoptosis, metastasis and epithelial mesenchymal transition (EMT), which complements the proposed function of LSM1 in mRNA regulation and provides a putative mechanism for LSM1‐mediated tumour progression.^7^, ^8^ Thus, LSM1 was found to be an important gene in maintaining the transformation phenotype of cancer cell lines.

Genomic instability is a molecular genetic marker for a variety of tumours. As the main form of genome instability, gene amplification plays an important role in the occurrence and development of many human malignant tumours.^1^, ^9^ LSM1 is a member of the LSm family of RNA‐binding proteins and a key member of the LSm1‐7 complex. Overexpression of LSM1 may play a role in pre‐mRNA splicing by mediating U4/U6 snRNP formation, affecting cell metabolism, cell cycle and destabilization of certain tumour suppressor transcripts in multiple ways, leading to cellular oncogenesis.^8^, ^10^ Increased expression of LSM1 may play a role in cellular transformation and the progression of several malignancies, including lung, mesothelioma and breast cancer. Selectively spliced transcript variants of this gene have been observed, and the pseudogene was located on the short arm of chromosome 9.^4^, ^7^, ^11^

Drug development is a complex and lengthy process, and requires significant human and financial resources to find more effective drug candidates. Gene expression profiling microarrays can simultaneously observe the expression status of thousands of genes in different individuals, tissues and developmental stages, and perform drug screening based on the differential expression of genes under different conditions, which can provide directions for drug development and accelerate the discovery and application of potential drugs.^1^ Therefore, in this study, the expression profile of LSM1 gene in breast cancer was mined and analysed through a multi‐omics strategy to analyse the biologic pathways and targets of drug candidates obtained by pharmacogenomic screening. The molecular mechanisms were further investigated to accelerate the drug discovery and development of breast cancer.

## 2 MATERIALS AND METHODS

### 2.1 Breast cancer cell lines and cell culture

Normal breast cells (H‐184B5F5/M10) and breast cancer cell lines (MDA‐MB361, MDA‐MB‐231, MDA‐MB‐453, MDA‐MB‐468, HS578T, ZR781, T47D and MCF7 (all cell lines were purchased from bioresource collection and research centre, Hsinchu, Taiwan) were used and cultured in medium supplemented with 10% foetal bovine serum in a humidified atmosphere with 95% air and 5% CO_2_ at 37°C, while the MDA‐MB cell line did not require CO_2_ conditions.

### 2.2 Real‐time PCR detection

RNA isolation of all samples was performed using EasyPrep Total RNA Kit (BIOTOOLS Co., Ltd.), as indicated above. Next, 1 μg of total RNA was reverse transcribed using a ToolScript MMLV RT kit. (BIOTOOLS Co., Ltd.) in a Applied Biosystems™ (ABI 7500) under the following reaction conditions: 65°C for 5 min, 42°C for 60 min and 70°C for 10 min. The resulting cDNAs were subjected to quantitative real‐time PCR (qRT‐PCR) analysis using a TOOLS 2X SYBR qPCR Mix (BIOTOOLS Co., Ltd.) in a StepOnePlus Real‐Time PCR system. The conditions used included an initial step at 95°C for 10 min, followed by 40 cycles at 95°C for 15 s and a final step at 60°C for 1 min. Ct values were calculated using U6 (RNU6‐1) as reference. Untreated samples were used as controls to determine the relative fold changes in mRNA expression.

### 2.3 cBioPortal database

The relationships between LSM1 variants and the mutational landscape of LSM11 were retrieved from the cBioPortal for Cancer Genomics,^12^ which is a web platform of gene‐based data exploration. This public database includes 50,000 genes affecting the survival of 32 cancers, and we use this tool for survival analysis, mutations, copy number changes and overall survival (OS) of common differentially expressed genes (DEGs).

### 2.4 Oncomine database

ONCOMINE was used to analyse the difference in LSM1 expression between normal and BRCA tissue samples. In the ONCOMINE analysis, the screening criteria were set as cancer type breast cell carcinoma; gene LSM1; data type mRNA; analysis type cancer vs. normal analysis; thresholds: *p*‐value <1E‐4, fold change >1.5, gene rank top 10%. Student *t*‐tests were performed to detect differences between the normal tissue group and the BRCA group. In addition, a meta‐analysis of gene expression data was performed on ONCOMINE.^13^

### 2.5 bc‐GenExMiner database

The Breast Cancer Gene Expression Miner (bc‐GenExMiner) database is a web‐based application that provides an estimate of prognostic value and is based on 21 public datasets.^14^ In this study, the bc‐GenExMiner database was used to identify LSM1 expression associated with a subset of breast cancers and to estimate the prognostic significance of LSM1 based on the different oestrogen receptor and subtypes status in breast cancers.

### 2.6 GEPIA2 database

GEPIA2 is based on gene expression analysis of tumour and normal samples from TCGA and GTEx databases.^15^ This study analysed the alteration, survival map and gene expression level of LSM1 in BRCA through this database.

### 2.7 TIMER database

In this study, we analysed seven hubs of expression of genes in BRCA associated with tumour purity and abundance in their immune infiltration (B cells, CD4+ T cells, CD8+ T cells, neutrophils, macrophages and dendritic cells (DC)). The prediction accuracy is further corroborated using 3809 transcriptional profiles available elsewhere in the public domain. In addition, we explored the relationship between the number of genetic copies of variation and the abundance of immune infiltrates.^16^ This database was used to analyse the correlation of LSM1 involvement in immune infiltration in breast cancer in order to fully explore the immunological, clinical and genomic characteristics of the tumour.

### 2.8 Q‐omics drug dabase

The drug sensitivity profiling based on LSM1 expression was analysed using the CRISPR‐screen data repository of the GDSC algorithm in Q‐omics v.1.0 (accessed on 12 January 2022).^17^ The cell line analyses available to Q‐omics are as follows: (1) cross‐association analyses between any pair of datasets according to gene expression, mutations, shRNA screening data, sgRNA screening data and drug screening data; (2) change (induction) analyses of gene expression before/after drug treatments; and (3) scatter/box plot analyses of pairs according to gene expression, mutations, shRNAs, sgRNAs and drugs.

### 2.9 Clinical data source and survival analysis

LinkedOmics contains multi‐omics data from 32 TCGA cancer types and a total of 11,158 patients with primary tumours, including mutations, copy number alterations (CNAs), methylation, mRNA expression, mutation data at the expression site level at the miRNA gene level and clinical data. We downloaded the TCGA dataset of breast cancer mRNA and screened 1093 clinical cases containing LSM1 gene expression, and ranked the cases in the top 50% and bottom 50% of expression levels as the high and low expression groups, with a test standard of *p* < 0.001. A total of 20,051 genes were detected, and the Pearson correlation between each gene expression level and LSM1 expression level was performed using an online analysis tool, and the 50 genes with positive correlation with LSM1 expression and the highest correlation coefficient were selected for gene heatmap.^18^

### 2.10 Human BRCA tissue microarray and specimens

Tissue microarray (TMA) slides (CBA4) containing human breast cancer, metastatic and normal tissues were purchased from SuperBioChips Laboratories. A immunohistochemistry (IHC) was performed as described in a previous report.^19^ We then used Taiwan Biobank specimens and data collected between 2008 and 2016. The Taiwan Biobank data source contains information on basic demographics, medical history and genotype. All clinical studies were performed in accordance with the approved guidelines of the Show Chwan Memorial Hospital Institutions Review Board (IRB: 1080604). Informed consent was obtained from all patients involved in this study.

### 2.11 Small‐interfering RNA transfections

For siRNA transfections, cancer cells were transfected with 10 nM siRNAs using Lipofectamine 3000 and analysed 24 h post‐transfection. LSM1 siRNAs were purchased from OriGene Co (SR309058).

### 2.12 Cell migration and invasion assay

The migration and invasion assay were performed as described.^20^

### 2.13 Statistical analysis

Each mRNA experiment was performed at least 3–6 times, and all data are represented as mean ± standard error of the mean (S.E.M.) of the quadruplicate measurements. The statistical significance was evaluated using a two‐way analysis of variance (anova) test using GraphPad Prism 8.0 (GraphPad Software). The differences were considered significant when *p* < 0.05.

## 3 RESULTS

### 3.1 Analysis of genome‐wide variants in BRCA

**FIGURE 1:**
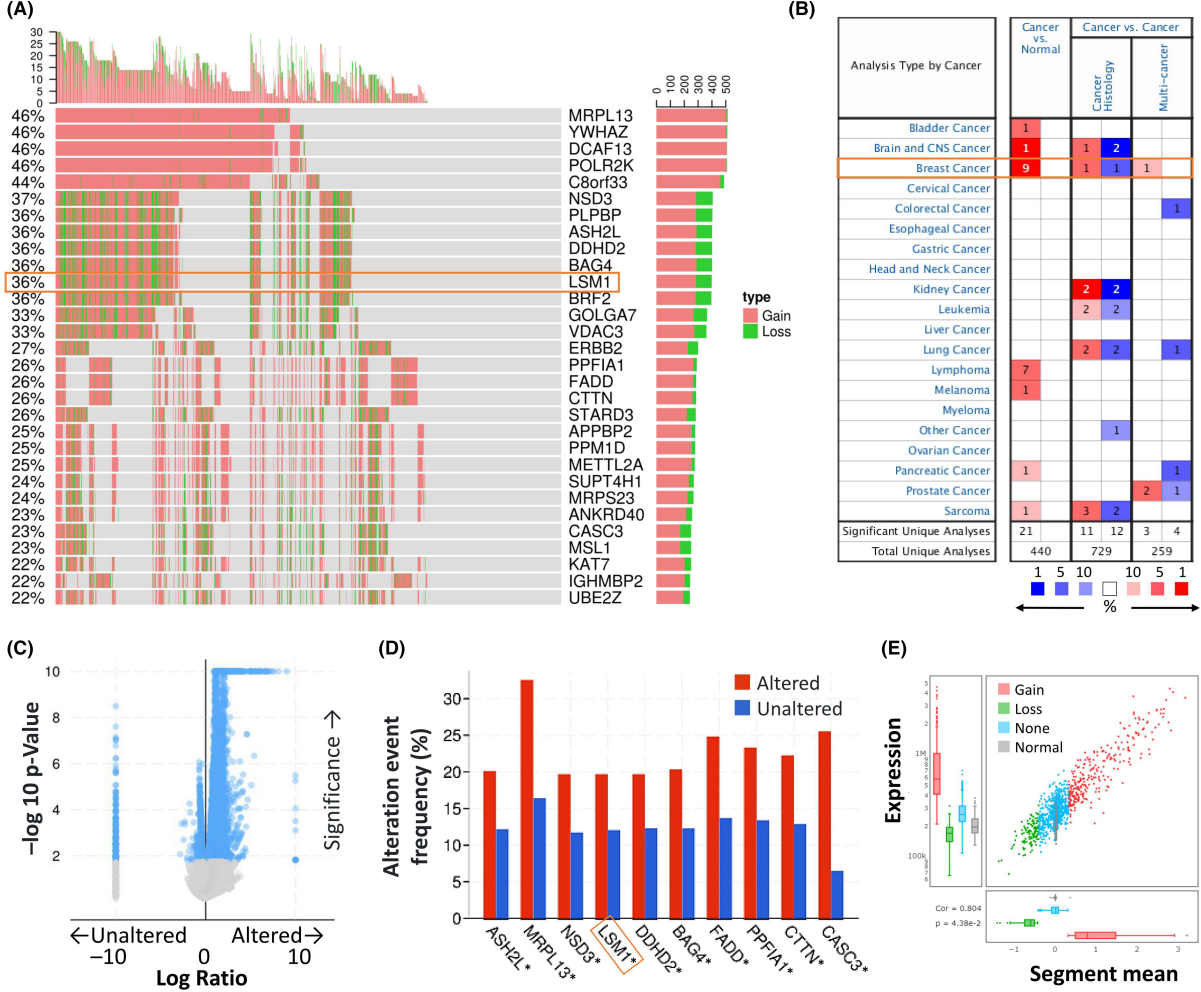
LSM was significantly overexpressed in breast cancer compared with normal breast tissue. (A) Waterfall plot illustrates the relations between the top 30 genes and the CNV in cancer patients for a specific breast cancer. (B) The mRNA expression levels of LSM1 in multiple cancers on ONCOMINE database. Transcriptional expression of LSM1 was significantly high in breast cancer. (C) Volcano plots exhibiting genes associated with alterations in LSM1 CNA frequency. (D) The top 10 genes with the highest alteration frequencies were markedly enriched in the altered group. (E) The distribution and correlation of CNV in breast cancer were marked with red (gain) and green (loss) to visualize the distribution of log_2_ ratios

The copy number variation (CNV) data from BRCA patients were processed by the TCGA database and clinical information is shown in Figure 1. The top CNV genes were MRPL13, YEHAZ, DCAF13, POLR2K (46%), C8orf33 (44%), NSD3 (37%), PLPBP, ASH2L, DDHD2, BAG4 and LSM1 (36%; Figure 1A). We found that LSM1 was also highly variable and further analysed by the Oncomine database, and the expression of LSM1 gene in pan‐cancer was overexpressed in the breast cancer tissue compared to normal breast tissue (Figure 1B). We also analysed the frequency of co‐occurrence of gene alterations with LSM1 gene alterations (Figure 1C) and found a total of 852 genes in which gene alterations co‐occurred in breast cancer. However, ASH2L, MRPL13, NSD3, LSM1, DDHD2, BAG4, FADD, PPFIA1, CTTN, CASC3 alterations and non‐alterations were among the most frequently mutated cohorts of gene alterations (Figure 1D). In addition, significant changes in LSM1 gain and loss were observed in CNV ratio distributions and box plots (Figure 1E).

### 3.2 The Genetic Alteration Landscape of LSM1 in breast cancer

**FIGURE 2:**
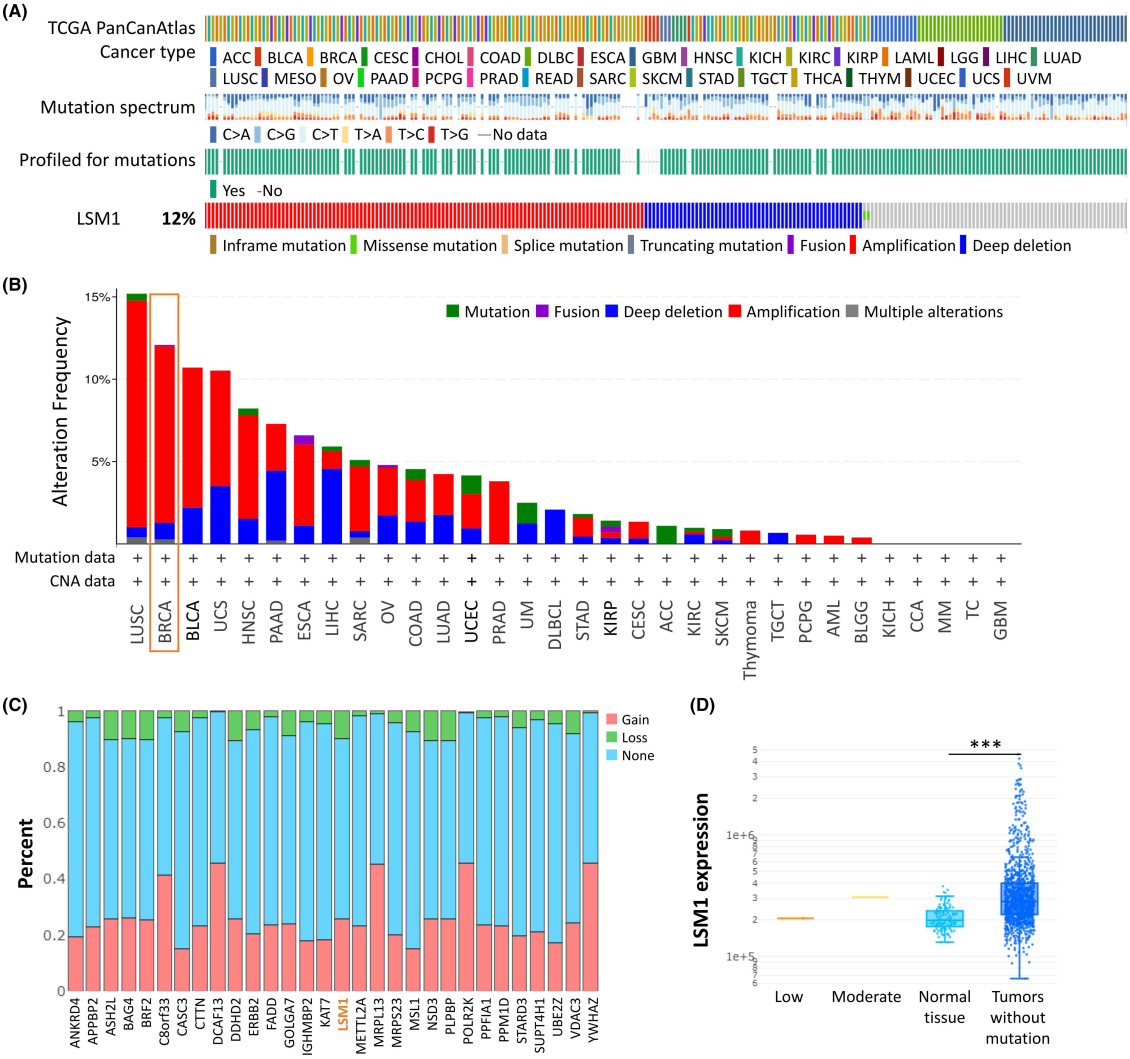
LSM1‐related transcription factor alternation analysis in breast cancer. (A) LSM1 gene mutation frequencies and types in breast cancer samples. (B) Genetic alterations of LSM1 gene in various cancer types using cBioPortal cancer genomics analysis. (C) Gene mutation frequencies of LSM1 in various carcinoma types. The red bars indicate gene amplifications, blue bars are homozygous deletions, green bars are non‐synonymous mutations, and grey bars indicate multiple alterations. (D) The expression of LSM1 in different types of mutant tumour tissues (*n* = 990). ***p < 0.001

We then used cBioPortal to determine the type and frequency of LSM1 alterations based on whole‐exome sequencing data from the BRCA in TCGA. We found that the LSM1 gene was mutated in up to 12% of all cancers (Figure 2A). We then investigated the genetic alterations of LSM1 in various tumour types in the TCGA dataset. We found that BRCA tumour samples had the next highest frequency of LSM1 genetic alterations (Figure 2B). To investigate the relationship between mutation frequency and LSM1, we first examined the expression of many representative genes from each of the major LSM1 pathways. We observed the gene expression levels of LSM1 master regulators in each tumour (Figure 2C). We then analysed the correlation between the mutations of LSM1 and TMB/MSI in BRCA from TCGA. The median TMB in the MSI group was significantly higher than that in the MSS group. The median TMB of the LSM1‐positive group was statistically higher than that of the LSM1‐negative group (Figure S1). We further investigated the relationship between LSM1 expression and BRCA mutation type. The results showed significant differences between normal tissues and tumours without mutations (Figure 2D).

### 3.3 Survival and expression of LSM1 in BRCA tissues and normal tissues

**FIGURE 3:**
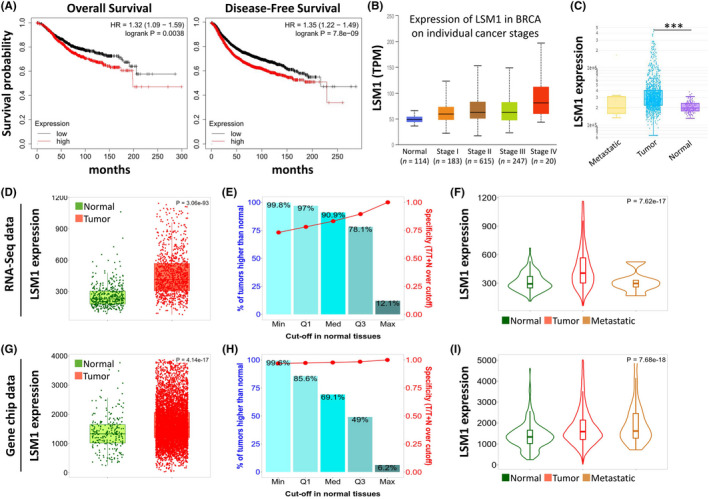
Relative expression and survival of LSM1 in BRCA tissues based on multiple databases. (A) Overall and disease‐free survival estimates for LSM1 mRNA levels from Kaplan–Meier plotter database. (B) Box plot to evaluate LSM1 mRNA expression in BRCA patients based on pathological stage. (C) LSM1 expression in normal, BRCA primary tumour and metastatic tumour (*n* = 990). Boxplots (D and G), bar charts (E and H) and violin plots (F and I) of LSM1 gene expression from RNA‐sequencing data and Gene chip data. ***p < 0.001

We analysed the effect of LSM1 on overall survival and disease‐free survival in BRCA patients using the Kaplan–Meier plot. We found that high LSM1 expression was associated with poor prognosis (Figure 3A and Figure S2). Then, we assessed LSM1 expression according to different clinical stages, and we found that LSM1 expression was significantly increased in both tumour tissues and patients with advanced stages (Figure 3B,C). We used TNM plot to analyse LSM1 expression from gene microarray data and RNA‐seq data (Figure 3D,G) (*p* = 3.06e−93, *p* = 4.14e−17). We also analysed the sensitivity and specificity of LSM1 in BRCA, and the results show the percentage of tumour samples showing higher expression of the selected gene than normal samples at each major cut‐off value. Example outputs for normal tumour comparisons were shown in Figure 3E,H. LSM1 mRNA expression also correlated significantly with cancer stage, with patients with advanced cancer tending to express higher LSM1 mRNA expression (Figure 3F,I).

### 3.4 LSM1 upregulation accelerates the biological features of breast cancer

**FIGURE 4:**
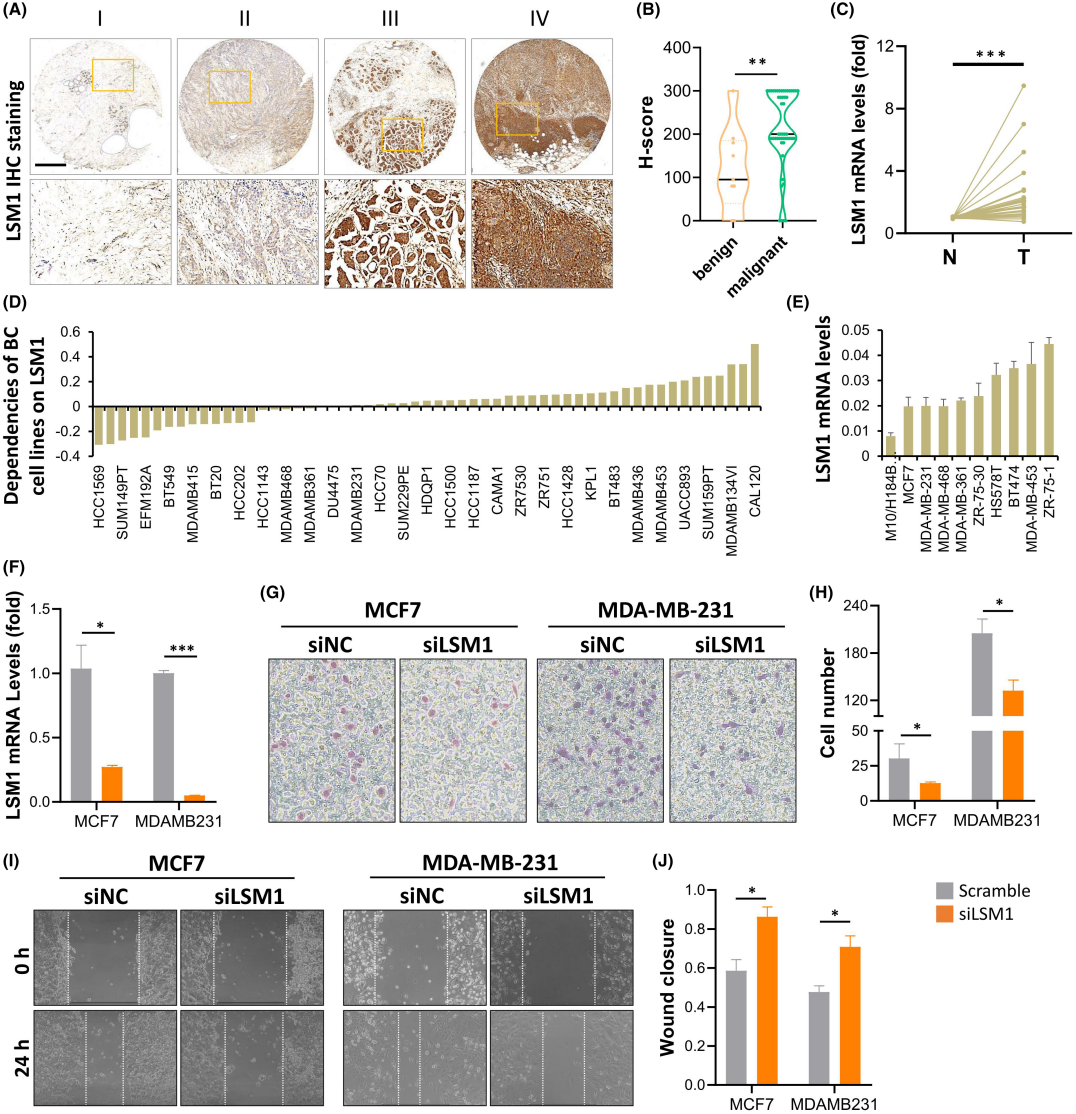
LSM1 promotes tumour progression in breast cancer. (A) Representative images of LSM1 expression in breast cancer tissues at different staining stages. (B) The expression levels of LSM1 in breast cancer were assessed in benign and malignant violin plots. (C) qPCR analysis of LSM1 in 30 paired BRCA and non‐tumour tissues. N and T represent non‐tumour and tumour tissues, respectively. (D) Significance of dependency of LSM1 in 57 BRCA cell lines based on the CRISPR screen. (E) mRNA expression of LSM1 in normal breast cells and multiple breast cancer cells. (F) The qPCR revealed LSM1 expression was significantly decreased in siLSM1 group breast cancer cells. (G and H) Wound healing assays of MCF7 and MDA‐MB‐231 cell lines. (I and J) Transwell assays of MCF7 and MDA‐MB‐231 cell lines were used to determine the invasion of BRCA cells. *p < 0.05, **p < 0.01, ***p < 0.001. Scale bar = 500 μm

We evaluated LSM1 detected in tumour tissues using a commercial breast tissue microarray (TMA) using immunohistochemistry. The results of LSM1 expression in breast cancer tissues in IHC staining are shown in Figure 4A. The H‐score of LSM1 increased significantly with tumour progression (Figure 4B). Next, we examined the mRNA expression of LSM1 in 30 paired BRCA and non‐tumour tissues from Taiwan biobank. The qPCR results showed that LSM1 was significantly upregulated in BRCA tissues (Figure 4C). We further analysed the dependence of 57 breast cancer cell lines on LSM1 and mapped the LSM1 dependence (fold change in sgRNA abundance relative to control transfected cells) of breast cancer cell lines, which were ranked by increasing LSM1 dependence (Figure 4D). We also confirmed the mRNA levels of LSM1 in breast cancer cells and normal breast cells (H‐184B5F5/M10), and the results were consistent with the datasets data, where LSM1 levels were significantly higher in breast cancer cells than in normal breast cells (Figure 4E). To further confirm the impact of LSM1 on breast cancer tumorigenesis, the endogenous expression level of LSM1 was knocked down with siLSM1 transfection in MCF7 and MDA‐MB‐231 cell lines (Figure 4F). Next, to demonstrate the role of LSM1 in the context of BRCA development, we performed cell migration and invasion assays after knockdown of LSM1 in MCF7 and MDA‐MB‐231 cell lines. The results showed that the wound healing assay confirmed that LSM1 knockdown led to inhibition of migration of both MCF7 and MDA‐MB‐231 breast cancer cells (Figure 4G,H). In addition, LSM1 deficiency also led to a slowing of invasion of both breast cancer cells (Figure 4I,J). Collectively, these results suggested that LSM1 can act as a tumour enhancer via promoting the migration and invasion of BRCA cells.

### 3.5 Correlation of LSM1 with clinicopathological parameters in breast cancer

**FIGURE 5:**
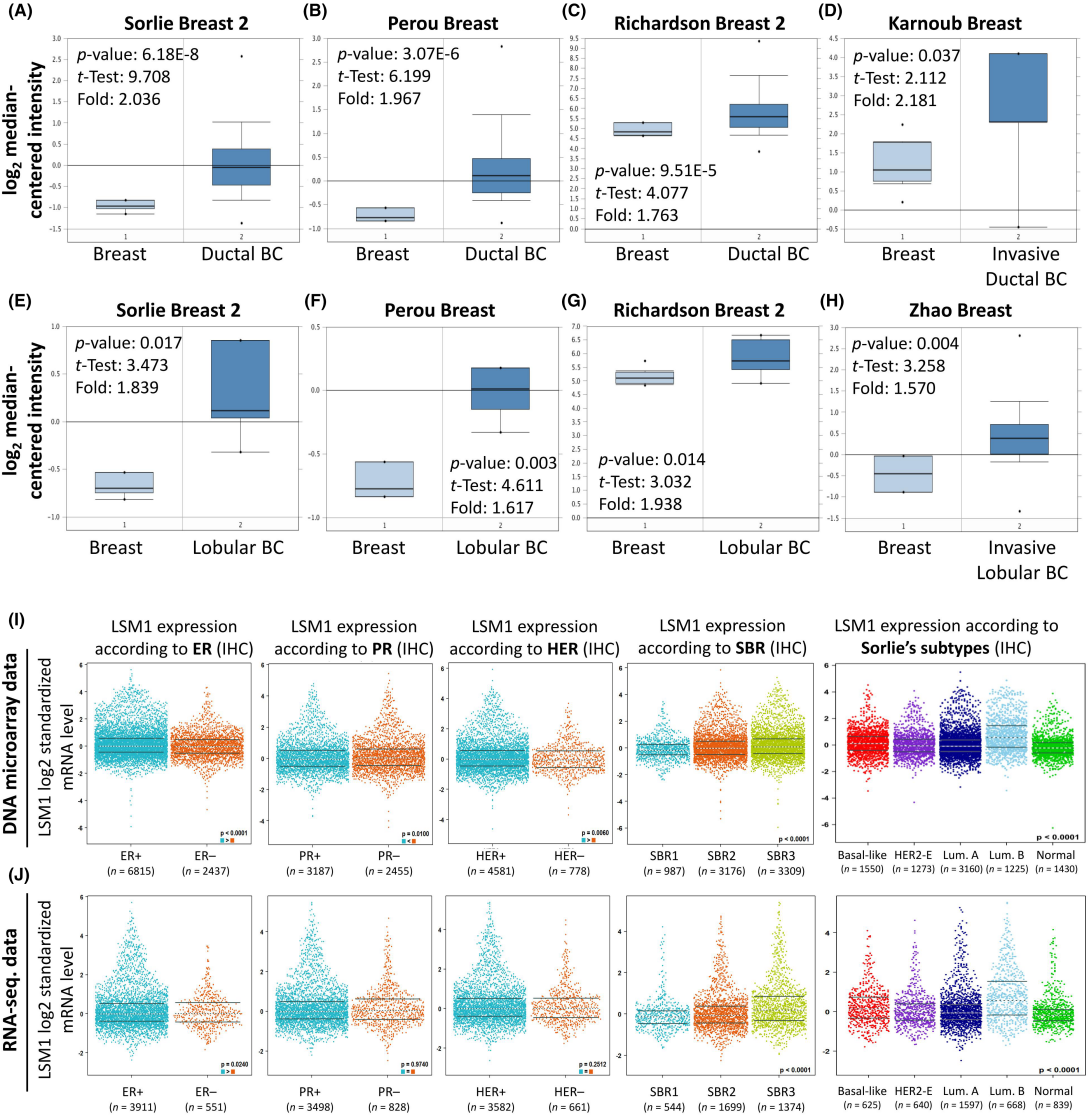
Box and whiskers plots and differential expression of in breast cancer patients based on different kinds of classified parameters. LSM1 mRNA levels from (A and E) Sorlie Breast 2 Statistics cohort, (B and F) Perou Breast Statistics cohort, (C and G) Richardson Breast 2 Statistics cohort, (D) Karnoub Breast Statistics cohort, (H) Zhao Breast Statistics cohort in BRCA and normal tissue. Note: *p* < 0.05 indicates statistical significance; LSM1 was among the top 10% overexpressed genes in all five different datasets of BRCA. (I and J) LSM1 mRNA expression levels were shown in breast cancer patients by bee swarm in DNA microarray datasets and RNA‐sequencing datasets. (ER, oestrogen receptor; PR, progesterone receptor; HER2, human epidermal growth factor receptor 2)

To validate the role of LSM1 in breast cancer, we verified the expression of LSM1 in different types of breast cancer using the Oncomine dataset. The results showed that LSM1 mRNA levels were significantly higher in ductal breast cancer (Figure 5A–C), invasive ductal breast cancer (Figure 5D), lobular breast cancer (Figure 5E–G) and invasive lobular breast cancer (Figure 5H) compared to matched normal tissues. Overall, our findings suggest that LSM1 upregulation is highly associated with breast cancer and that LSM1 plays an important role in tumour cancer progression. To evaluate the correlation between LSM1 expression and BRCA clinicopathological parameters, we performed an analysis by bc‐GenExMiner datasets. Both DNA microarray (Figure 5I) and RNA sequencing data (Figure 5J) confirmed a high expression of LSM1 mRNA in ER+ (ER+ > ER−, *p* < 0.0001). In addition, LSM1 mRNA expression was significantly lower in the PR− group (ER+ < ER−, *p* = 0.01) compared to the progesterone receptor (PR+) in the DNA microarray database. LSM1 mRNA expression was significantly upregulated in the oestrogen receptor (HER2+) group compared to the corresponding HER2− group (HER+ > HER−, *p* = 0.006) in the DNA microarray database. Analysis of the Scarff Bloom & Richardson equivalence state (SBR) criterion showed that increased SBR levels were associated with elevated LSM1 transcript levels significantly correlated (SBR1 < SBR2 < SBR3, *p* < 0.0001) in DNA microarray and RNA sequencing data. The results in different breast cancer subtypes also showed that the expression of LSM1 was lower in normal tissues than in other subtypes. Taken together, the above results demonstrate the prognostic value of clinicopathological parameters in breast cancer.

### 3.6 Functional network analysis of the predictive LSM1 gene

**FIGURE 6:**
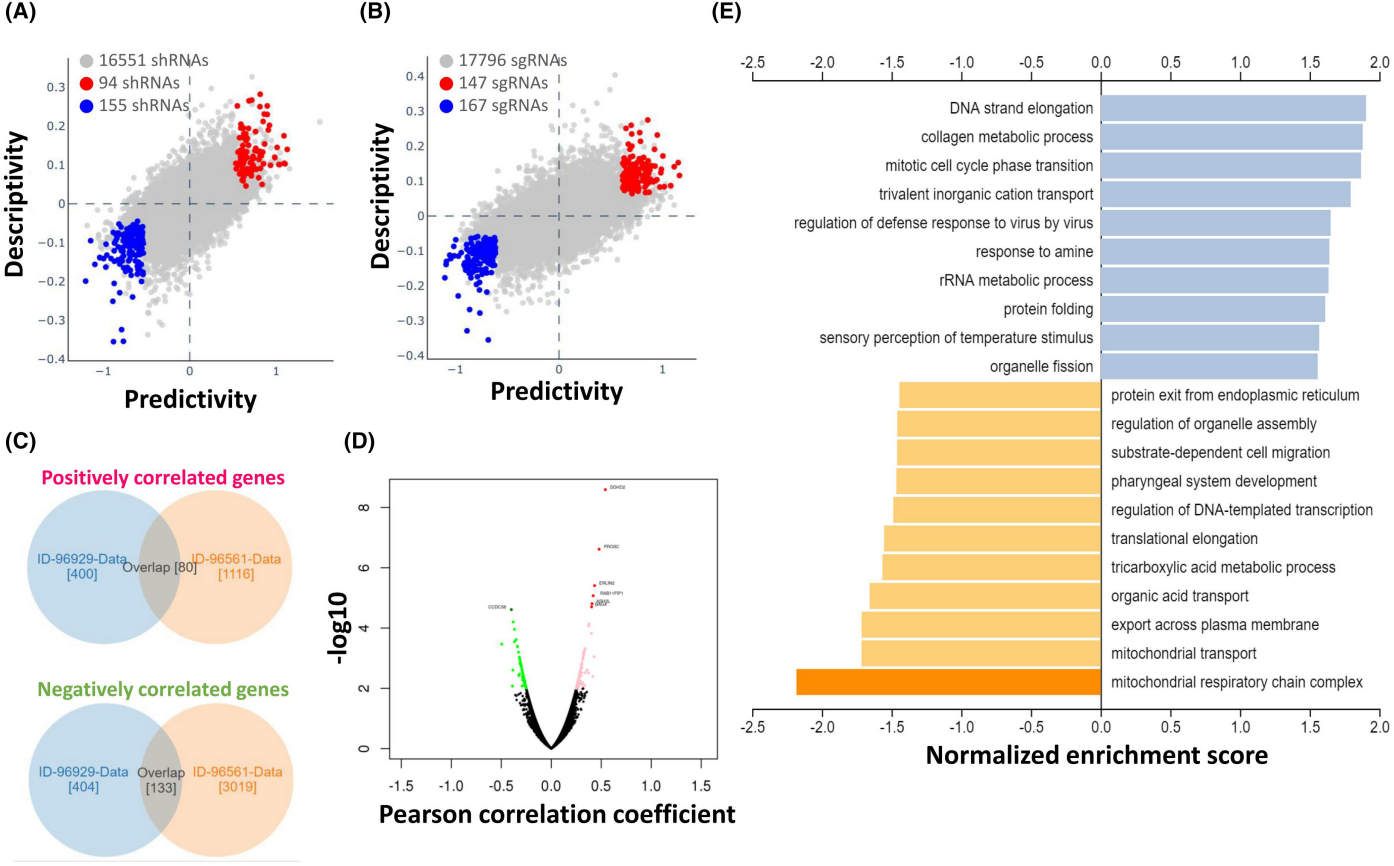
Functional prediction and enrichment analysis of LSM1 expression in breast cancer. The predictability and descriptiveness between mRNA expression and shRNA (A) and sgRNA (B) functions are plotted with breast cancer cell lines. (C) Genes with shRNA/sgRNA overlap are identified in the positive correlation and negative correlation Venn diagram analysis. (D) Volcano plot showing Pearson's test analysis of differential gene expression associated with LSM1 in BRCA. (E) Functional enrichment analysis of LSM1 in BRCA

We further analysed the association between LSM1 shRNA/sgRNA efficacy and target gene expression levels in different breast cancer cell lines. We attempted to calculate two‐way predictive and descriptive scores for each of the more than 16,000–17,000 genes using statistical tests. We further verified the association between particular immune cell contents and overall survival in BRCA patients by Q‐omics analysis (Figure 6A,B). Among the shRNA potencies, 94 genes (red circles in Figure 6A) showed positive scores in predictiveness and descriptiveness, while 155 genes (blue circles in Figure 6A) showed negative scores. Similarly, among the sgRNA potencies, 147 genes (red circles in Figure 6B) showed positive scores in terms of predictiveness and descriptiveness, while 167 genes (blue circles in Figure 6A) showed negative scores. To further explore the potential functions and molecular pathways of the LSM1 gene in BRCA, we used the LinkedOmics database to identify LSM1 co‐expressed genes in the data of 975 patients from TCGA. A total of 7966 LSM1‐associated genes were altered, reflecting the important impact of the core gene LSM1 on the pathogenesis of BRCA. We then used Venn diagrams to evaluate the differentially expressed genes (DEGs) with positive and negative overlap in Figure 5A,B (Figure 6C). These clusters of genes positively associated with LSM1 were shown as red dots in the volcano plot, while the clusters of genes negatively associated with LSM1 were indicated as green dots (*p* < 0.01 and FDR <0.01, Figure 6D). The top 20 significant gene clusters positively and negatively associated with LSM1 were shown by functional enrichment (Figure 6E).

### 3.7 LSM1 is correlated with genetic and immune infiltration level in BRCA

**FIGURE 7:**
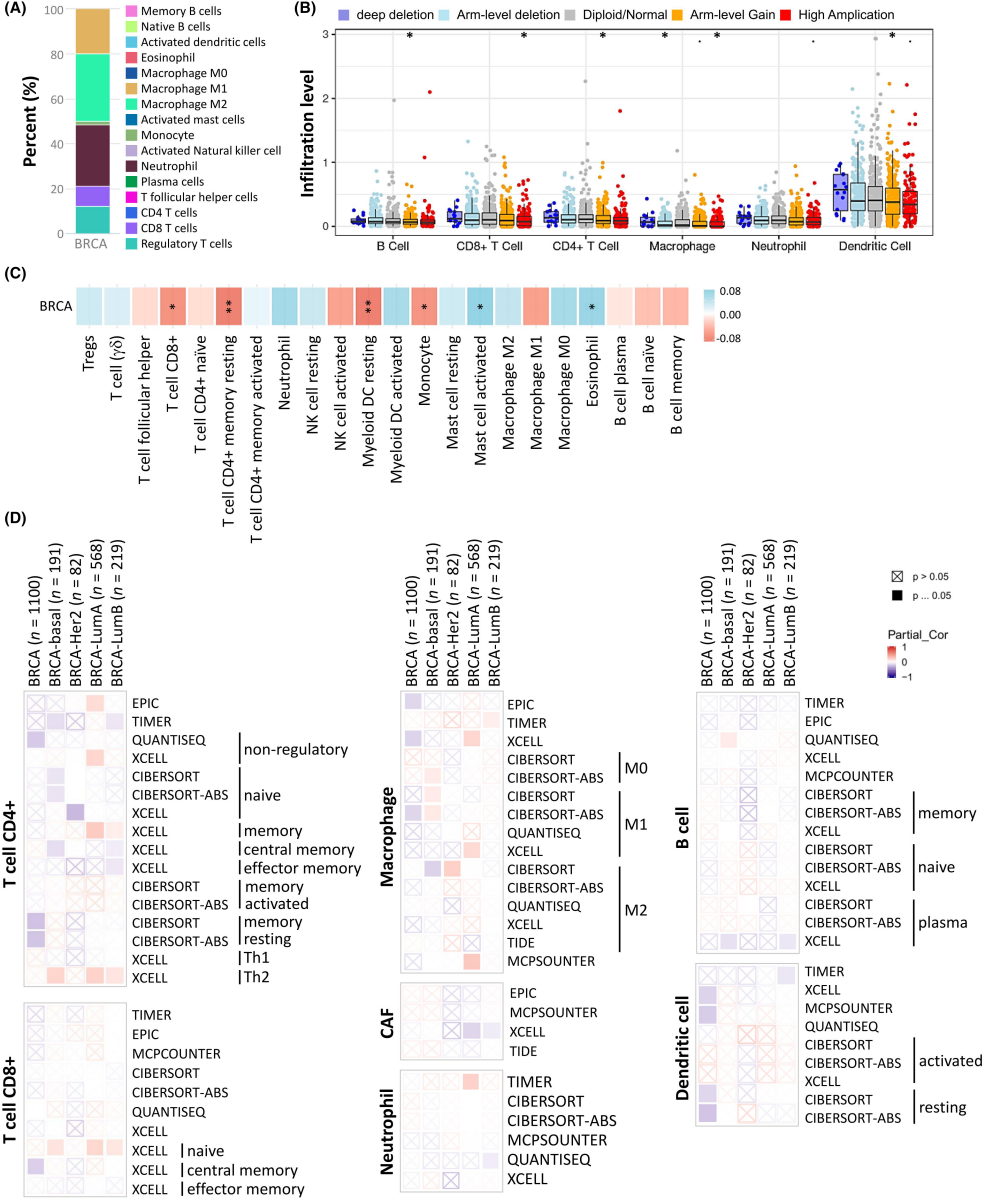
Correlations between the CNV of LSM1, immune cell infiltration, and prognosis in BRCA. (A) Immune cell bars show the expression of the LSM1 gene. (B) LSM1 copy number variable (CNV) affects infiltration levels of CD8 + T cells, macrophages, neutrophils and dendritic cells in BRCA. (C) Heatmap showing the correlation between LSM1 expression and immune infiltration in BRCA. (D) Correlation between the tumour‐immune microenvironment and LSM1 expression. *p < 0.05

We investigated the potential correlation between LSM1 expression in breast cancer and several mutations commonly seen in breast cancer and showed the correlation between LSM1 expression and six mutations (Figure S3A) from the TIMER dataset. The values adjacent to the highly mutated genes were the distribution of genetic variants between the driver mutation (red) and not‐mutated (grey) samples. We analysed the effect of LSM1 mutations on immune cell infiltration in various cancer types and the effect of immune cell type in pan‐cancer by mutation module. The effect of LSM1 mutations on immune cell infiltration in pan‐cancer was analysed by PIK3CA and TP53 mutation modules, and the effect of immune cell types in pan‐cancer (Figure S3B,C). The results showed that LSM1 expression was significantly reduced in mutated PIK3CA (*p* = 0.017); however, LSM was not affected by mutated TP53. This also suggests that the association of LSM1 with immunity may be related to PIK3CA mutations in BRCA (Figure S3D,E). Tumour‐infiltrating lymphocytes (TILs) play a key role in pan‐cancers, including breast carcinoma. In Figure 7A, it was shown that LSM1 regulates the different immune cells in breast cancer cells, with macrophages M1, M2 and neutrophil accounting for the highest percentage of immune cells. In addition, we further investigated the association between the CNV of LSM1 and immune cell infiltration in the prognostic model. The results showed that the absence or expansion of other forms of copy number may differentially modulate the infiltration of immune cells in breast cancer compared to normal copy number (Figure 7B). We additionally investigated the correlation between different immune cells and LSM1 expression in breast cancer using different algorithms. LSM1 expression was negatively correlated with T cell CD8+, T cell CD4+ memory resting, Myeloid DC resting and Monocyte (Figure 7C). We further evaluated the relationship between LSM1 and a variety of tumour‐infiltrating immune cells. After multiple database predictions, we hypothesized that LSM1 has the potential to modulate immune responses. Therefore, we further expanded the analysis of the significant correlation between LSM1 and different levels of immune cell infiltration. It was worth noting that the expression of LSM1 was highly negatively correlated with the level of infiltration of CD8+ T cells and CD4+ T cells. We comprehensively screened the LSM1 profiles of all tumours and used different algorithms to analyse the expression of each cancer type and correlate it with LSM1 expression levels (Figure 7D and Figure S4). Increasing information shows that immune cell infiltration can accelerate tumour progression and recurrence and affect immunotherapy and clinical outcome. Correlation between LSM1 in BRCA expression, abundance of immune infiltrates (B cells, T cells, macrophages, neutrophil, dendritic cells) and survival time are shown in Figure S4. BRCA patients with low LSM1 gene expression and high B cell and neutrophil infiltrates had a longer survival time than patients with high gene expression and low macrophage infiltrates (*p* < 0.01). BRCA‐LumB patients with high LSM1 gene expression and high B cell, T cell and dendritic infiltrates tended to have a longer survival time than those with high LSM1 gene expression and low macrophage and neutrophil infiltrates (*p* < 0.01; Figure S5). In this study, we found a significant correlation between the survival time and the expression of LSM1 in the infiltration of the five immune cell types—B cells, neutrophils, CD4+ T cells, macrophages and myeloid dendritic cells—indicating that LSM1 expression in combination with immune cell status can predict prognosis. Further research is needed to explore the potential immune therapy using LSM1.

### 3.8 Pharmacogenetic screening for potential drugs that inhibit LSM1

**FIGURE 8:**
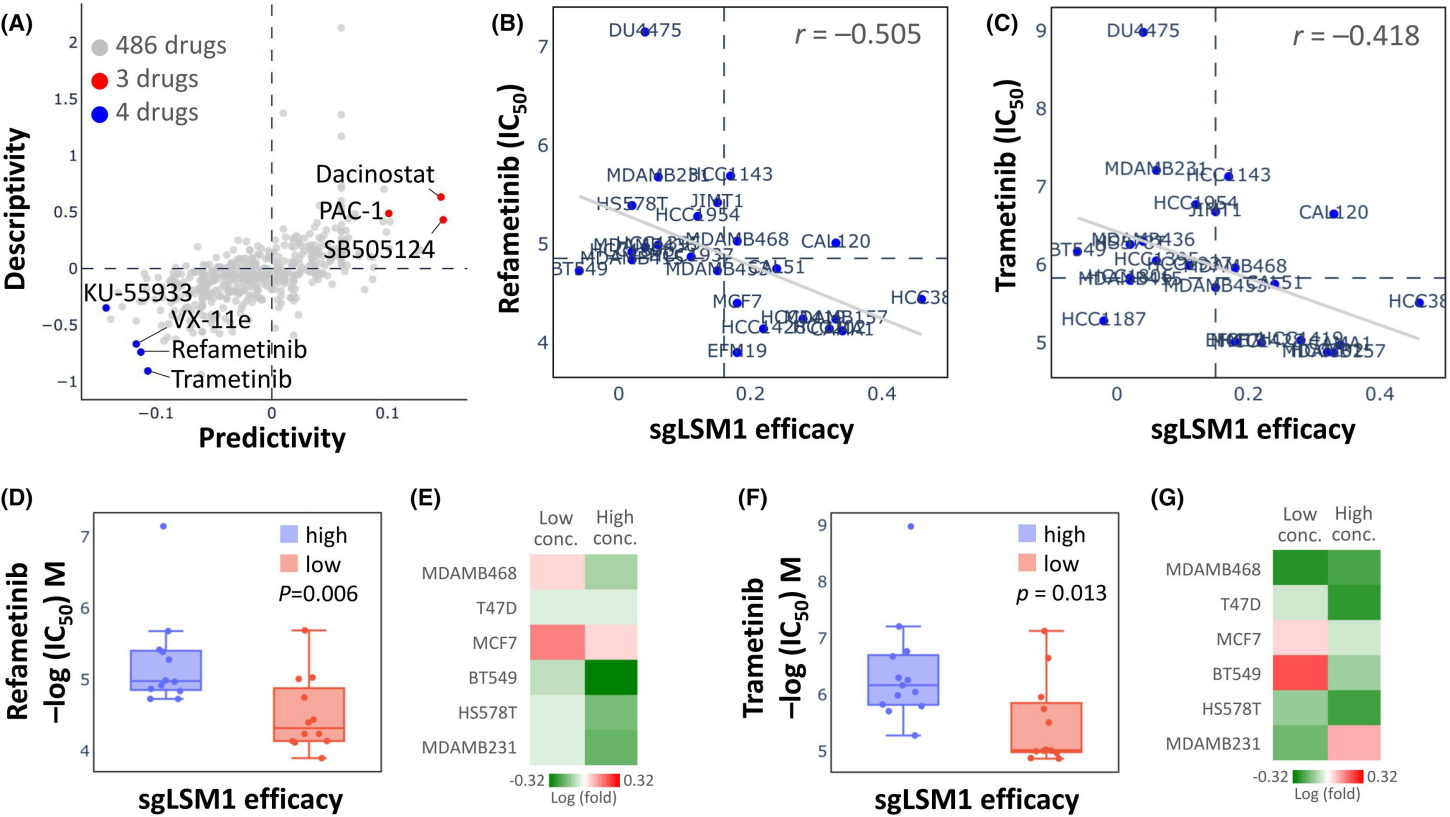
Drug sensitivity and cytotoxicity analysis in breast cancer cells. (A) Use of the database to query LSM1 gene signatures and screen for potential drugs. (B and C) Drug sensitivity of sgLSM1 gene to refametinib and trametinib in BRCA cell lines. (D and F) LSM1 efficacy of refametinib and trametinib in inhibiting breast cancer cells. (E and G) Drug sensitivity of different doses of refametinib and Trametinib in treating different breast cancer cells

We further retrieved the LSM1 gene library from the pharmacogenetic database to find potential drugs for the treatment of BRCA. Since the Q‐omics database contains gene signatures from drug‐treated or shRNA/sgRNA‐transfected cancer cell lines,^21^ it can be used to explore the association between drugs and knockdown/knockout. As shown in Figure 8A, 7 of the 486 drugs were of significant interest, including KU‐5933, VX‐11e, Refametinib and Trametinib, which inhibited LSM1 overexpression. When queried in the Q‐omics database for the relationship between Refametinib and Trametinib for knockout LSM1 co‐expression gene features, we found a high sensitivity and negative correlation between Refametinib and Trametinib for CRISPR LSM1 knockout (Refametinib: *r* = −0.505; Trametinib: *r* = −0.418; Figure 8B,C). Thus, Refametinib and Trametinib have anti‐cancer potential to inhibit the growth of breast cancer cells with high expression of LSM1. The efficacy of Refametinib and Trametinib in knockout LSM1 on both Refametinib and Trametinib drugs was significant in inhibiting the overexpression of LSM1 (Figure 8D,F). To validate the bioinformatics analysis, six BRCA cell lines (MDA‐MB‐468, T47D, MCF7, BT549, HS578T and MDA‐MB‐231) were used to test the efficacy of Refametinib and Trametinib on breast cancer cells. The results showed that high concentrations of Refametinib were more effective in inhibiting six breast cancer cell lines (Figure 8E); furthermore, Trametinib was effective in inhibiting breast cancer cells at both low and high concentrations (Figure 8G). We also performed the sensitivity of both drugs to LSM1 by MDA‐MB‐231 cell line and the results were similar to (Figure 8 and Figure S6).

## 4 DISCUSSION

Genomic instability is a molecular genetic marker for a variety of tumours. As the main form of genome instability, gene amplification plays an important role in the occurrence and development of many human malignant tumours.^1^, ^9^ LSM1 is a member of the LSm family of RNA‐binding proteins and a key member of the LSm1‐7 complex. Overexpression of LSM1 may play a role in pre‐mRNA splicing by mediating U4/U6 snRNP formation, affecting cell metabolism, cell cycle and destabilization of certain tumour suppressor transcripts in multiple ways, leading to cellular oncogenesis.^8^, ^10^ Increased expression of LSM1 may play a role in cellular transformation and the progression of several malignancies, including lung, mesothelioma and breast cancer. Selectively spliced transcript variants of this gene have been observed, and the pseudogene was located on the short arm of chromosome 9.^4^, ^7^, ^11^

Not only cancer genetics but also aberrant epigenetic changes have been reported to be involved in the tumorigenesis and progression of BRCA.^22^ While LSM1 was first reported to be overexpressed in breast cancer, the copy number of chromosome 8p11‐12 region was increased.^23^ LSM1 overexpression in MCF10A cells leads to non‐dependent proliferation of IGF and induces the production of soluble factors that can substitute for insulin action without activating the IGF‐I pathway. We report for the first time that LSM1 expression levels are associated with PIK3CA mutations, but are not regulated by TP53 mutations. Moreover, previous studies have shown that LSM1 is highly associated with PIK3CA and BCL‐2 in regulating the chemotherapy resistance pathway in small cell lung cancer and ‘IGF‐1 receptor/EGFR synergy in lung cancer’, suggesting that LSM1 plays an important role with PIK3CA in lung cancer tumour progression.^24^ Therefore, we speculate that high expression of LSM1 may be associated with mutations in the PIK3CA gene leading to uncontrolled cell division and recovery, which in turn affects the significant elevation of LSM1 in breast cancer.

Tumour immune/inflammatory cell infiltration was an indicator of the host immune response to cancer cells.^25^, ^26^, ^27^ We hypothesized that since the LSM1 cluster network was rich in cancer and inflammation/immune related pathways, their high expression levels in a variety of cancers may be associated with tumour immune infiltration. To this end, we investigated the association between LSM1 expression and tumour immune infiltration in different datasets by multi‐omics. We found that T cell CD8+, T cell CD4+ memory resting, myeloid DC resting and monocyte infiltration were negatively correlated with LSM1 expression in the breast cancer infiltrate cohort. This suggests that in addition to disease prognosis, LSM1 may also reflect immune status. This observation was consistent with our observations in the pathway enrichment analysis of the LSM1 positive and negative correlation clustering network. Thus, these findings not only suggest that LSM1 was involved in the immune invasion of breast cancer, but also provide a new window for monitoring the tumour immune microenvironment and may serve as a potential prognostic biomarker for the immune response to these cancers. Therefore, the results of this study may have clinical implications for the prognostic evaluation and follow‐up management of immunotherapy.

In pharmacogenetic analysis, refametinib and trametinib treatment simulated the effects of LSM1 inhibition on breast cancer cell lines and reduced breast cancer cell growth at both high concentrations. Refametinib showed potent anti‐proliferative activity in vitro in each of the HCC cell lines evaluated and also in xenograft and allograft models. Refametinib either alone or in combination has the ability to modulate MEK1 expression as it is a repressor of MEK1/2 in different cancers. A positive effect on metastatic spread can be achieved with sorafenib monotherapy and combination therapy. When used in combination, refametinib and sorafenib act synergistically in multiple models to reduce tumour growth and prolong survival.^28^, ^29^, ^30^, ^31^ In addition, trametinib has been reported to inhibit the growth of ERRα and KRAS‐mutant lung cancer in different cancers.^32^, ^33^ Although reduced TNFα production was observed in vivo, the combination therapy activated CD8+ T cell‐mediated immunity and increased survival in an immunoreactive mouse model carrying glioma.^34^ Therefore, the development of potential refametinib and trametinib as agents to reduce the high expression of LSM1 and thereby slow the progression and metastasis of BRCA is a future therapeutic goal.

In this study, we analysed the value of LSM1 mRNA expression in breast cancer patients in relation to its diagnosis and prognosis using TCGA data. Multi‐omics analysis revealed that LSM1 mRNA expression was significantly higher in breast cancer tissues and correlated with several clinical parameters, such as high expression in ER and HER‐positive patients. Moreover, our analyses of LSM1 indicated statistical correlations of LSM1 expression with clinical prognosis, genetic alteration, tumour immune infiltration, tumour microenvironment, immune checkpoint molecules and immune cells pathway, helping to understand its role in BRCA from the perspective of clinical tumour samples. Since the present study only analysed the data of LSM1 transcript level, it did not involve the study of LSM1 protein level. Therefore, further experimental validation was still needed to explore the molecular mechanisms associated with LSM1 in BRCA. Finally, we identified that refametinib and trametinib potentially inhibit the overexpression of LSM1 in breast cancer cells by pharmacogenomic screening of appropriate drugs and by testing different cell lines. Therefore, targeting the LSM1 signalling axis may provide a dual role of gene suppression and immunotherapeutic response in breast cancer.

## AUTHOR CONTRIBUTIONS

**Yen‐Dun Tony Tzeng:** Formal analysis (equal); investigation (equal); methodology (equal); validation (equal); writing – original draft (supporting). **Kuan‐Hao Tsui:** Data curation (equal); visualization (equal). **Ling‐Ming Tseng:** Conceptualization (equal); resources (equal). **Ming‐Feng Hou:** Conceptualization (equal); investigation (equal); resources (equal). **Pei‐Yi Chu:** Conceptualization (equal); investigation (equal); resources (equal). **Jim Jinn‐Chyuan Sheu:** Data curation (supporting); formal analysis (supporting); software (equal). **Chia‐Jung Li:** Data curation (equal); formal analysis (equal); funding acquisition (equal); software (equal); supervision (equal); writing – review and editing (lead).

## CONFLICT OF INTEREST

The authors declare that they have no conflict of interest.

## Supporting information


Data S1

## Data Availability

The data sets used for the current study are available from the corresponding author upon reasonable request.
