# Supplementary material for: Integrated analysis of pivotal biomarker of LSM1, immune cell infiltration and therapeutic drugs in breast cancer
Source: J Cell Mol Med. 2022 Jun 12;26(14):4007–20. doi: 10.1111/jcmm.17436 (PMC9279588; doi:10.1111/jcmm.17436)
Supplement: Supplementary file 1 — Data S1 [file JCMM-26-4007-s001.docx]

Supplementary Figure S1


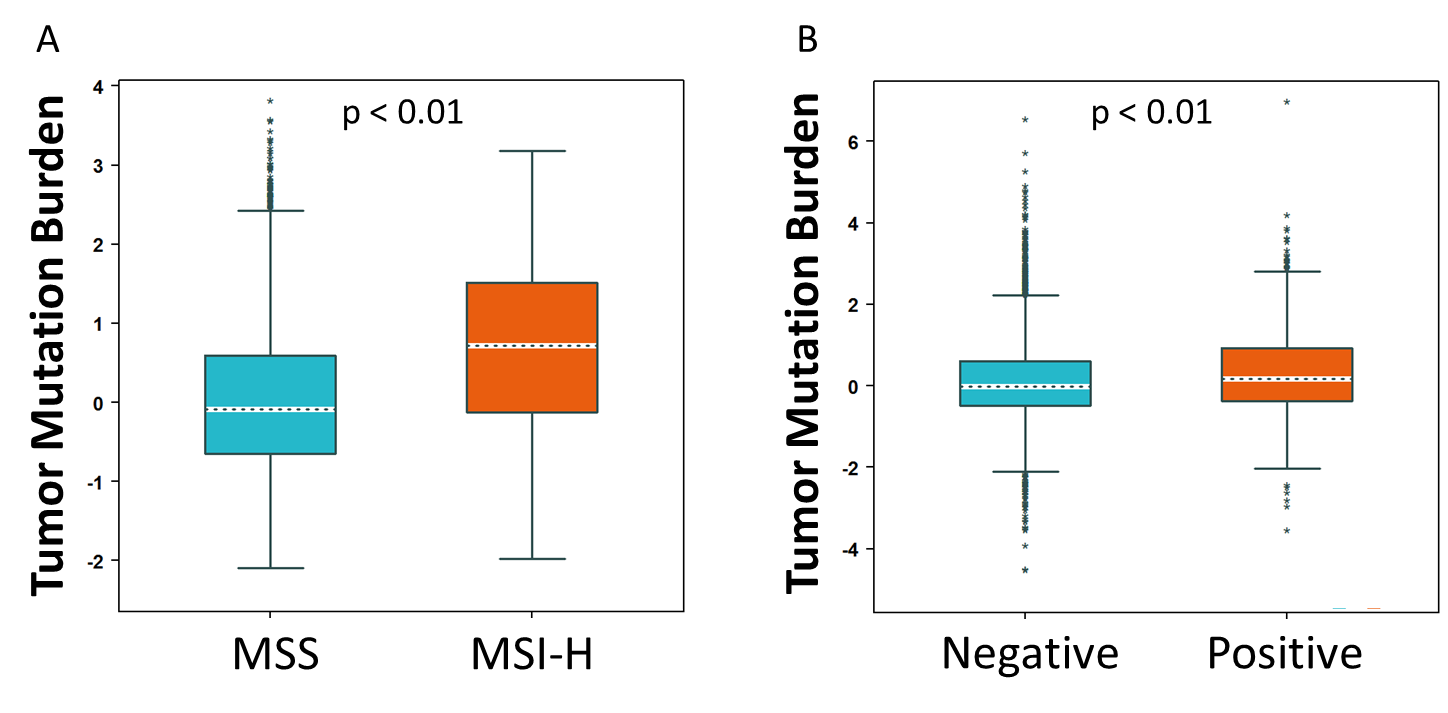


Supplementary Figure S1. Boxplots of TMB according to MSI status **(A)** and LSM1 levels **(B)**. TMB, tumor mutational burden; MSI, microsatellite instability; MSS, microsatellite-stable.

Supplementary Figure S2


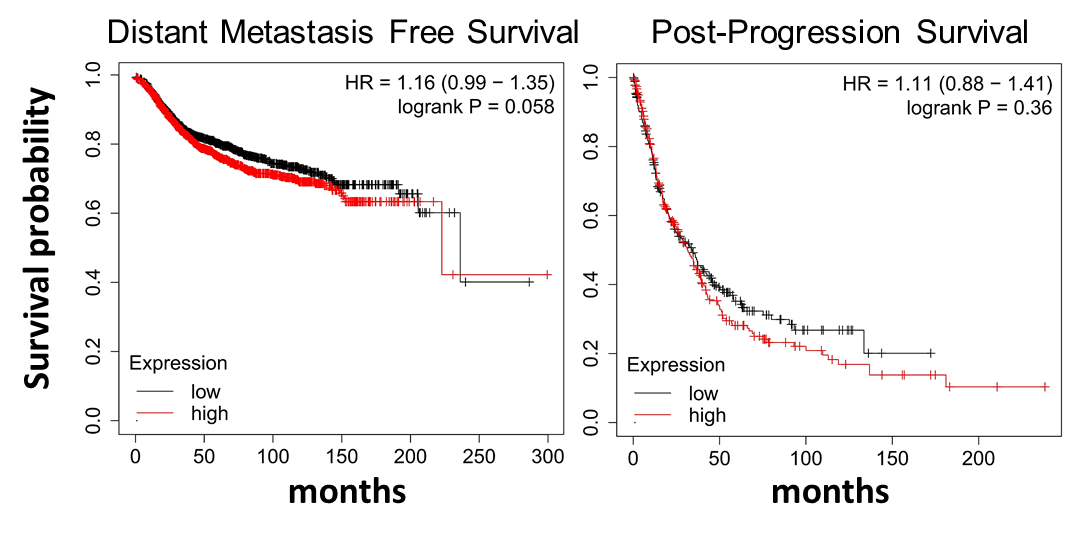


**Supplementary Figure 2:** The effect of LSM1 gene expression on tumor prognosis was studied with the Kaplan-Meier plotter. Kaplan-Meier plotter is used for breast cancer survival analysis.

Supplementary Figure S3


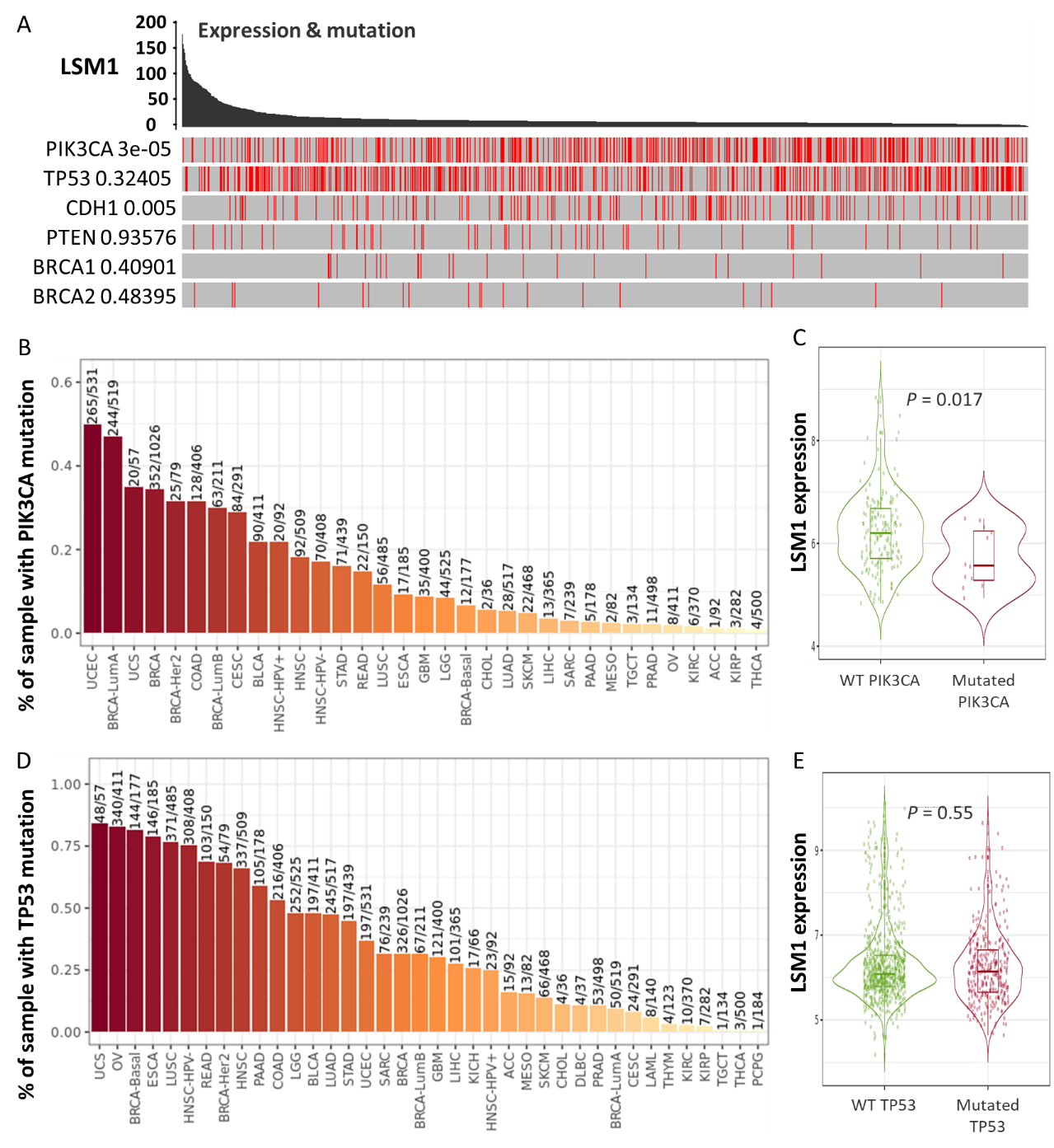


**Supplementary Figure 3:** TIMER dataset to assess the association between immune infiltration and genetic or clinical features. (A) The relationship between LSM1 and six highly mutated genes in breast cancer (red: mutated; gray: non-mutated). (B) The Gene Mutation module compares PIK3CA mutation status between LSM1 gene expression in pan-cancer. (C) Statistics of PIK3CA mutation status between LSM1 gene expression in breast cancer. (D) The Gene Mutation module compares TP53 mutation status between LSM1 gene expression in pan-cancer. (E) Statistics of TP53 mutation status between LSM1 gene expression in breast cancer (n = 1017).

Supplementary Figure S4


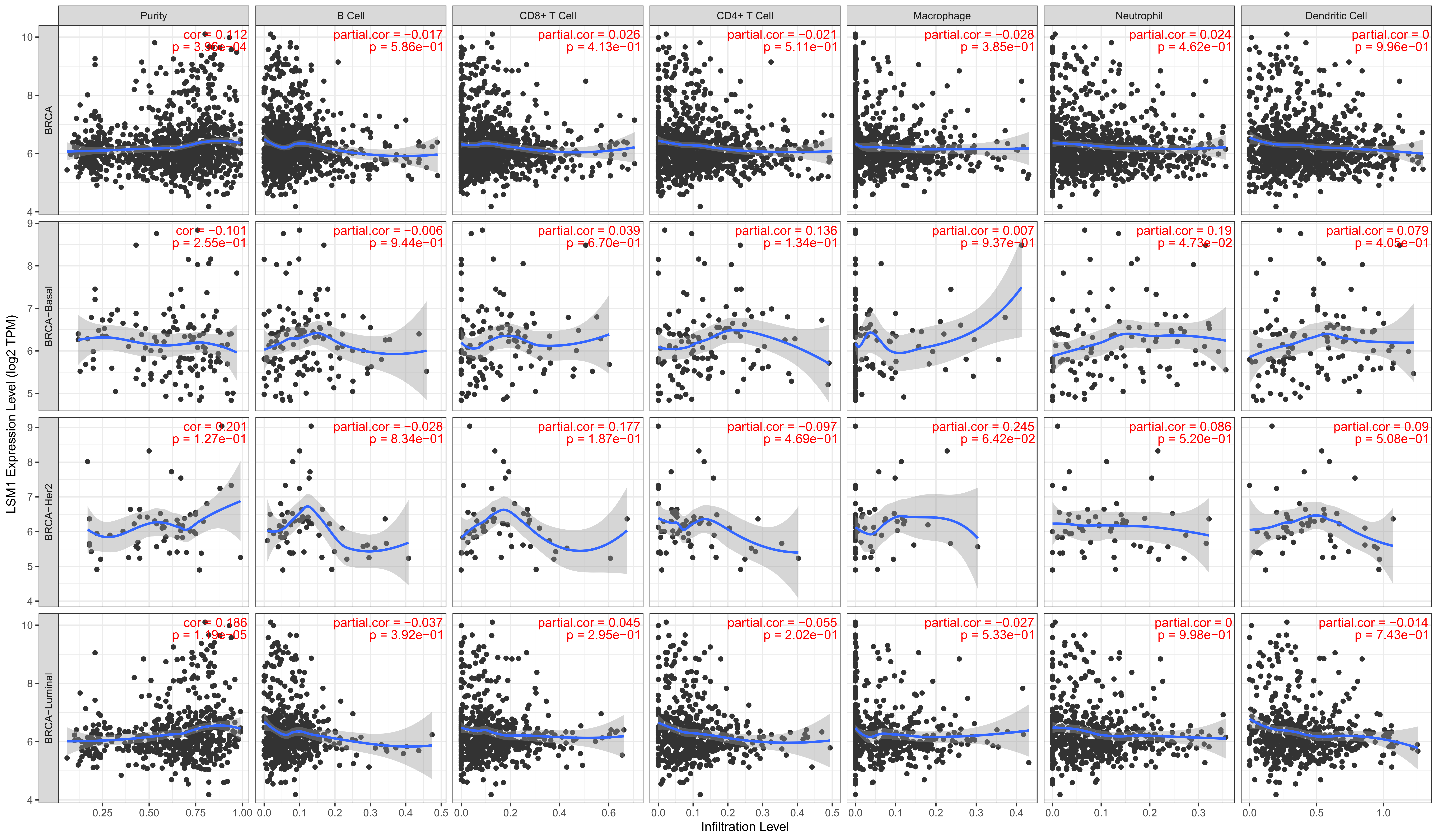


Supplementary Figure S4. Correlation analysis of LSM1 expression and in filtration levels of immune cells in BRCA tissues.

Supplementary Figure S5


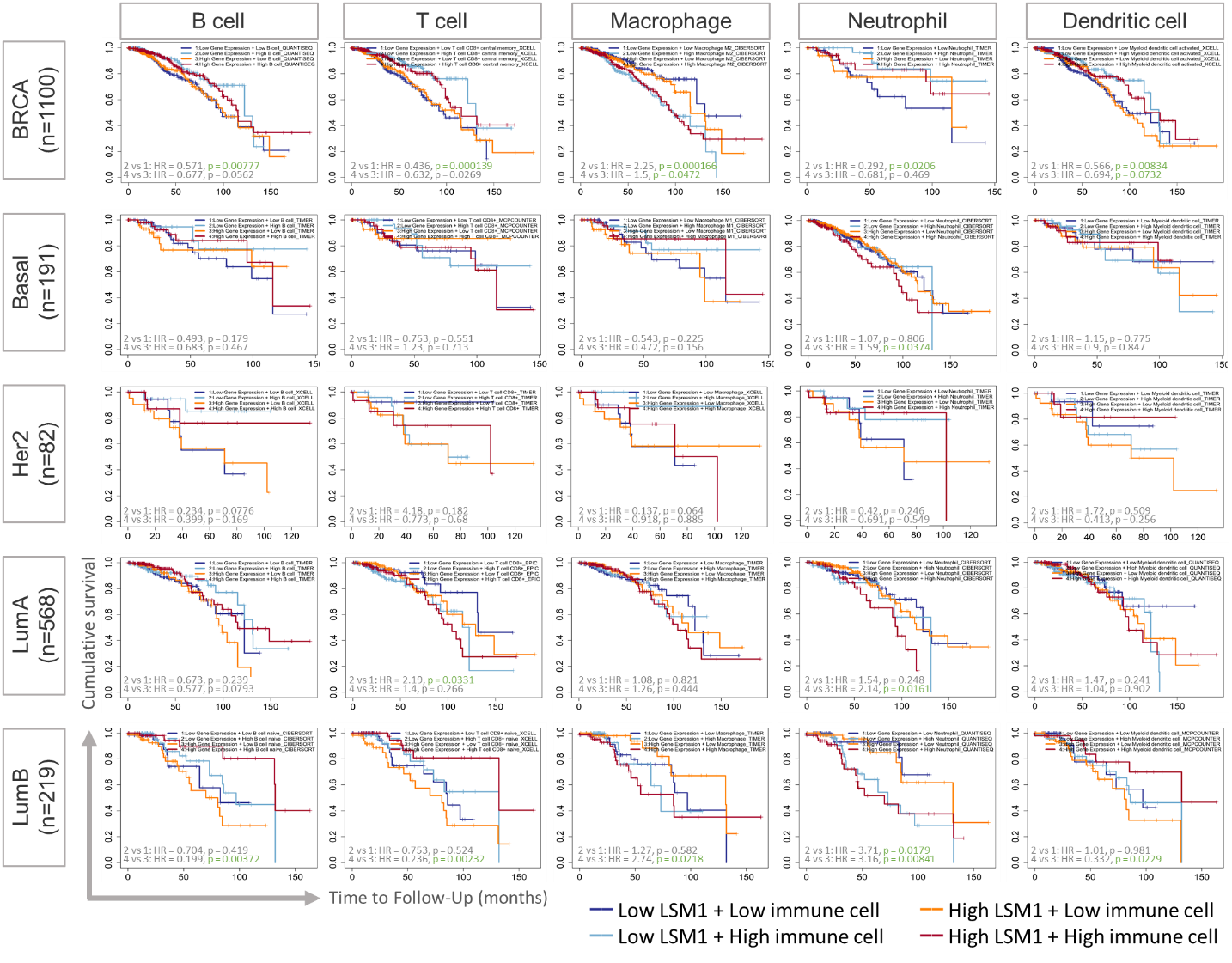


Supplementary Figure S5. Correlation of LSM1 expression with immune infiltration level in the TIMER database.

Supplementary Figure S6


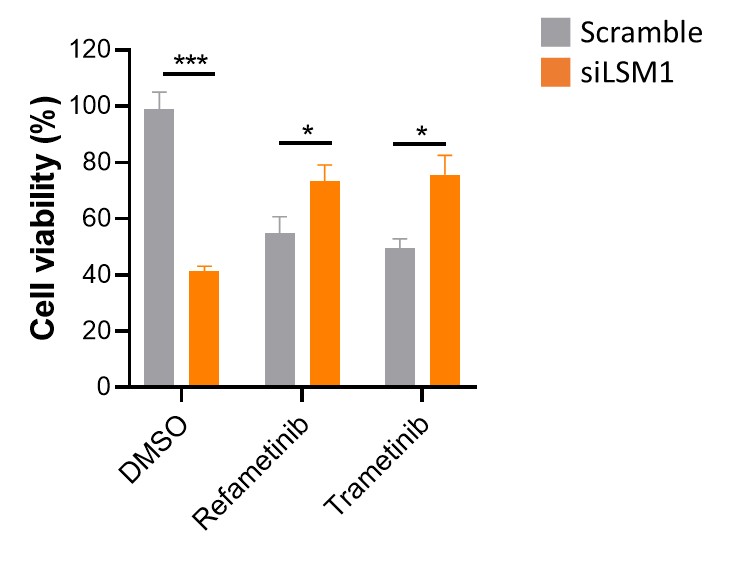


Supplementary Figure S6. Cell survival analysis Refametinib and Trametinib were treated for 24 hours in LSM1 gene deficient MDA-MB-231 cells. The IC50 doses of Refametinib and Trametinib were: 15 uM and 0.5 uM, respectively.
